# Comment on “Subureteral Injection with Small-Size Dextranomer/Hyaluronic Acid Copolymer: Is It Really Efficient?”

**DOI:** 10.1155/2018/4078183

**Published:** 2018-08-12

**Authors:** S. Arena, C. Romeo

**Affiliations:** Department of Human Pathology in Adult and Developmental Age “Gaetano Barresi”, University of Messina, Viale Gazzi, AUO “Gaetano Martino”, 98124 Messina, Italy

We read with great interest and appreciated very much the article written by Üre et al. [1]. In their study, the authors evaluated the efficiency of subureteral small-size (80-120 *μ*m) dextranomer/hyaluronic acid copolymer injection treatment in children affected by 1 to 4 grade vesicoureteral reflux, finding an overall success rate of 97% of the treated patients. Previously, Aydogdu et al. [2], comparing small-size dextranomer/hyaluronic acid copolymer and deflux (normal-size dextranomer/hyaluronic acid copolymer), showed no difference in effectiveness between these two agents with the advantage that small-size dextranomer/hyaluronic acid copolymer treatment was less expensive. It has been described that 80-250 *μ*m-diameter dextranomer microspheres in a stabilized hyaluronic acid gel recruited numerous myofibroblasts around the dextranomer particles, a foreign body inflammatory reaction with a high density in CD68 positive cells, stimulating an enhancement in collagenous stroma [3]. Moreover, it has been suggested that accumulation of fibrillar collagen and subsequent extracellular matrix after normal-size dextranomer/hyaluronic acid copolymer injection might explain the tissue contraction in refluxing ureteral ending, modifying the ureteral length-to-diameter ratio and promoting the resolution of vesico-ureteral reflux [3]. We believe that it could be interesting to also study the histological changes in ureteral endings after small-size dextranomer/hyaluronic acid copolymer injection, to verify if this substance has a similar effect of refluxing ureteral tissue. Finally, in our opinion, the data of Üre et al. [1] are very interesting and we would like to congratulate the authors on their splendid paper.
